# Dealing With BRCA1/2 Unclassified Variants in a Cancer Genetics Clinic: Does Cosegregation Analysis Help?

**DOI:** 10.3389/fgene.2018.00378

**Published:** 2018-09-11

**Authors:** Roberta Zuntini, Simona Ferrari, Elena Bonora, Francesco Buscherini, Benedetta Bertonazzi, Mina Grippa, Lea Godino, Sara Miccoli, Daniela Turchetti

**Affiliations:** UO Genetica Medica, Azienda Ospedaliero-Universitaria di Bologna Policlinico S.Orsola-Malpighi and Centro di Ricerca sui Tumori Ereditari, Dipartimento di Scienze Mediche e Chirurgiche, Universitá di Bologna, Bologna, Italy

**Keywords:** BRCA1, BRCA2, VUS, breast cancer, ovarian cancer, hereditary cancer

## Abstract

**Background:** Detection of variants of uncertain significance (VUSs) in BRCA1 and BRCA2 genes poses relevant challenges for counseling and managing patients. VUS carriers should be managed similarly to probands with no BRCA1/2 variants detected, and predictive genetic testing in relatives is discouraged. However, miscomprehension of VUSs is common and can lead to inaccurate risk perception and biased decisions about prophylactic surgery. Therefore, efforts are needed to improve VUS evaluation and communication at an individual level.

**Aims:** We aimed at investigating whether cosegregation analysis, integrated with a careful review of available functional data and *in silico* predictions, may improve VUSs interpretation and counseling in individual families.

**Methods:** Patients with Breast Cancer (BC) and/or Ovarian Cancer (OC) fulfilling established criteria were offered genetic counseling and BRCA1/2 testing; VUSs identified in index cases were checked in other relatives affected by BC/OC whenever possible. As an alternative, if BC/OC clustered only in one branch of the family, the parental origin of the VUS was investigated. Public prediction tools and databases were used to collect additional information on the variants analyzed.

**Results:** Out of 1045 patients undergoing BRCA1/2 testing in the period October 2011–April 2018, 66 (6.3%) carried class 3 VUSs. Cosegregation analysis was performed for 13 VUSs in 11 kindreds. Seven VUSs (53.8%) did not cosegregate with breast/ovarian cancer in the family, which provided evidence against their role in cancer clustering in those families. Among the 6 cosegregating VUSs, for two (BRCA1 c.5152+2T>G and BRCA2 c.7975A>G) additional evidence exists from databases and *in silico* tools supporting their pathogenicity, which reinforces the hypothesis they may have had a predisposing effect in respective families. For the remaining four VUSs (31%), cosegregation analysis failed to provide relevant information.

**Conclusion:** Our findings suggest that cosegregation analysis in a clinical context may be helpful to improve test result interpretation in the specific family and, therefore, should be offered whenever possible. Besides, obtaining and sharing cosegregation data helps gathering evidence that may eventually contribute to VUS classification.

## Introduction

In recent years, the increasing requests for BRCA testing have led to increased identification of patients carrying Variants of Unknown Significance (VUSs) in these genes. Several international consortia have been established with the aim of classifying VUSs; since functional assays for BRCA1 and 2, unlike other genes, are of limited availability and accuracy, classification mainly relies on multifactorial analysis, which requires that a large amount of data from multiple families is collected (Goldgar et al., 2004; Spurdle et al., 2012). This implies that a long time is frequently needed before a variant is conclusively classified. Therefore, in the Cancer Genetics Clinics, the detection of a VUS poses substantial challenges for counseling and managing patients. In fact, according to the widely adopted variants classification in 5 categories, class 3 VUS are those for which available evidence, if any, fails to significantly support either a pathogenic or a neutral significance (Plon et al., 2008; Lindor et al., 2012). For carriers of variants falling in this category, the same management as for probands with no BRCA variants detected is recommended, and predictive genetic testing in relatives is discouraged (Plon et al., 2008; Lindor et al., 2012). However, miscomprehension of VUS has been reported to be common among counselees and referring physicians (Richter et al., 2013) and several studies have consistently shown that risk perception is significantly greater in VUS carriers, if compared to patients with uninformative results, with a higher rate of prophylactic surgery undertaken or considered (Vos et al., 2011, 2012; Culver et al., 2013; Richter et al., 2013; Welsh et al., 2017).

Therefore, the ongoing international initiatives aimed at classifying VUSs should be paralleled by efforts to improve VUSs interpretation and communication at an individual level.

In particular, aim of this study was to investigate whether cosegregation analysis, integrated with a careful review of available functional data and *in silico* predictions, may improve VUSs interpretation and counseling in individual families.

## Patients and Methods

### BRCA1 and BRCA2 Testing

The Cancer Genetics Clinic in Bologna is one of the four Hubs of a Hub-and-Spoke Network established in 2012 in the Emilia-Romagna region (Northern Italy) with the aim of identifying and managing women at familial risk of breast and ovarian cancer.

In patients fulfilling criteria for BRCA testing according to the regional protocol (Servizio Sanità Pubblica and Regione Emilia-Romagna, 2016), informed consent was collected and a venous blood sample drawn during a genetic counseling session.

Genomic DNA was extracted from peripheral blood leukocytes using standard techniques. Complete sequence analysis of BRCA1 and BRCA2 genes was performed through Next Generation Sequencing technology using ION Torrent^TM^ Oncomine^TM^ BRCA Research Assay (Life Technologies). Manual libraries preparation was generated from 20 ng of DNA per sample according to the manufacturer’s instructions with barcode incorporation. Templates for DNA libraries were prepared using the Ion Personal Genome Machine (PGM) Hi-Q View OT2 200 Kit (Life Technologies) on the Ion One Touch 2 according to the manufacturer’s instructions. Sequencing of 24 samples multiplexed templates was performed using the Ion Torrent PGM on Ion 318 chips using the Ion PGM Hi-Q^TM^ View Sequencing Kit (Life Technologies) according to the manufacturer’s instructions. Data analysis was performed using Torrent Suite (5.6) applying Oncomine BRCA Research Germline workflow. Any variant (either pathogenic or of unknown significance) was confirmed by Sanger sequencing. Moreover, analysis of BRCA1 deletions/duplications was performed by Multiplex Ligation Probe Amplification (MLPA) using the P002 kit of MRC Holland (Amsterdam, Netherlands), and data were analyzed using Coffalyser.net software. Mutation nomenclature follows the general recommendations of the Human Genome Variation Society (HGVS): cDNA and protein numbering were based on the reference sequence ID NM_007294.3 and NM_000059.3, respectively.

### Variant Classification

All variants were evaluated through the retrieval of information in the following public databases: UMD^1^, BRCA Exchange^2^, ARUP Scientific Resource for Research and Education: BRCA Database^3^, ClinVar^4^, LOVD IARC^5^, LOVD3^6^. All databases were last accessed 17 May 2018.

### *In silico* Predictions

Potential cryptic splice sites and exonic splicing enhancers were investigated through Human Splicing Finder^7^, and ESEfinder 3.0^8^.

The evaluation of conservation of BRCA1/2 amino acids and related probability of pathogenicity was assessed according to the multiple-sequence alignments available on the Align GVGD Website^9^ (Tavtigian et al., 2006).

### Retrieval of Functional Data

The retrieval of results from functional assays for the VUS considered, if any, was made by querying the databases LOVD3^6^ and UMD^1^, and the recent neXtProt Cancer variant portal^10^ (Cusin et al., 2018).

### Cosegregation Analysis

The search for VUSs was extended to other relatives affected by BC or OC if they were available and consenting. As an alternative, when BC/OC clustering was observed in only one branch of the family, parental origin of the variant was defined by testing one or both parents, depending on their availability and willing. Quantitative cosegregation analysis was performed through the “Analyze my variant” website (Ranola and Shirts, 2017), which uses three different statistical methods: full-likelihood method for Bayes factors (FLB) (Thompson et al., 2003), co-segregation likelihood ratios (CSLR) (Mohammadi et al., 2009) and meiosis counting method (Jarvik and Browning, 2016).

## Results

### BRCA Test Results

From 1st October 2011 to 30th April 2018, 1045 index cases underwent BRCA testing at our center. Among those, 188 (18%) were found to carry pathogenic variants: 104 (55%) in BRCA1 and 84 (45%) in BRCA2. Among the remaining patients, 744 (71.2% of the total) had no variants detected, while 113 (10.8%) carried VUS. Among the VUS detected (96 in total), 33 are classified as class 2, 59 as class 3 and 4 as class 4 (**Tables 1**, **2**). Overall, a total of 66 probands (6.3% of those tested) carried class 3 VUS.

**Table 1:** Class 3 (“uncertain”) variants identified in the population under study.

BRCA1			BRCA2		
Nucleotide change	Predicted effect on protein	Number of families carrying the variant	Nucleotide change	Predicted effect on protein	Number of families carrying the variant
c.1397G > A	p.Arg466Gln	1	c.2755G > A	p.Glu919Lys	1
c.1912G > A	p.Glu638Lys	1	c.2944A > C	p.Ile982Leu	1
c.1934C > A	p.Ser645Tyr	1	c.3519T > G	p.Ile1173Met	1
c.3613G > A	p.Gly1205Arg	1	c.3749A > G	p.Glu1250Gly	1
c.3783A > T	p.Leu1261Phe	1	c.4291G > A	p.Ala1431Thr	1
c.3878C > T	p.Ala1293Val	1	c.4603G > T	p.Ala1535Ser	1
c.4013A > G	p.Lys1338Arg	1	c.476T > C	p.Val159Ala	1
c.4054G > A	p.Glu1352Lys	1	c.5200G > A	p.Glu1734Lys	1
c.4223A > G	p.Gln1408Arg	1	c.5390C > G	p.Ala1797Gly	1
c.441+5A > G		2	c.5498A > G	p.Asn1833Ser	1
c.457A > G	p.Ser153Gly	1	c.5702A > T	p.Glu1901Val	1
c.4777A > T	p.Ile1593Leu	1	c.5705A > C	p.Asp1902Ala	1
c.5509T > C	p.Trp1837Arg	4	c.5885T > C	p.Ile1962Thr	2
c.569C > T	p.Thr190Ile	1	c.599C > T	p.Thr200Ile	1
c.767G > A	p.Arg256Lys	1	c.6062A > G	p.His2021Arg	1
c.889A > C	p.Met297Leu	1	c.6290C > T	p.Thr2097Met	2
c.556T > G	p.Ser186Ala	1	c.7007+5G > A		1
c.1027_1028AA > TG	p.Asn343Cys	2	c.7534C > T	p.Leu2512Phe	1
c.2281G > C	p.Glu761Gln	1	c.7769C > T	p.Ser2590Phe	1
c.2589T > G	p.Val863 =	1	c.7786G > A	p.Gly2596Arg	1
c.3823A > G	p.Ile1275Val	1	c.8262T > G	p.His2754Gln	1
c.4895T > G	p.Val1632Gly	1	c.8351G > A	p.Arg2784Gln	1
			c.8386C > T	p.Pro2796Ser	1
			c.9006A > T	p.Glu3002Asp	1
			c.9458G > C	p.Gly3153Ala	1
			c.7756C > T	p.Leu2587Phe	1
			c.1244A > G	p.His415Arg	1
			c.1342C > T	p.Arg448Cys	1
			c.1550A > G	p.Asn517Ser	1
			c.1991G > A	p.Gly664Glu	1
			c.5386G > T	p.Asp1796Tyr	1
			c.8704G > A	p.Ala2902Thr	1
			c.9409A > T	p.Thr3137Ser	1
			c.9986A > G	p.Asn3329Ser	1
			c.9218A > C	p.Asp3073Ala	1
			c.6562A > G	p.Lys2188Glu	1
			c.1996A > G	p.Ile666Val	1

**Table 2:** Class 2 (“likely not pathogenic”) and class 4 (“likely pathogenic”) variants identified in the population under study.

	BRCA1			BRCA2			
	Nucleotide change	Predicted effect on protein	Number of families carrying the variant	Nucleotide change	Predicted effect on protein	Number of families carrying the variant	
**class 2**	c.522A > G	p.Gln174 =	1	c.1247T > G	p.Ile416Ser	2	**class 2**
	c.1137T > G	p.Ile379Met	1	c.1810A > G	p.Lys604Glu	1	
	c.1974G > C	p.Met658Ile	1	c.267G > A	p.Pro89Pro	2	
	c.2522G > A	p.Arg841Gln	1	c.5635G > A	p.Glu1879Lys	4	
	c.2883C > T	p.Asn961 =	1	c.6322C > T	p.Arg2108Cys	1	
	c.213-8A > C		2	c.68-7T > A		5	
	c.81-14C > T		1	c.7601C > T	p.Ala2534Val	1	
				c.3693T > C	p.Thr1231 =	1	
**class 4**	c.670+1G > A		1	c.8010G > A	p.Ser2670 =	1	
	c.4485-2A > C		1	c.8972G > A	p.Arg2991His	1	
	c.5017_5019delCAC	p.His1673del	15	c.9104A > C	p.Tyr3035Ser	1	
				c.9227G > T	p.Gly3076Val	1	
				c.9242T > C	p.Val3081Ala	1	
				c.9586A > G	p.Lys3196Glu	1	
				c.927A > G	p.Ser309 =	1	
				c.1395A > C	p.Val465 =	1	
				c.1514T > C	p.Ile505Thr	1	
				c.1820A > C	p.Lys607Thr	1	
				c.2817C > T	p.Thr939 =	1	
				c.4584C > T	p.Ser1528 =	2	
				c.6513G > T	p.Val2171 =	3	
				c.6927C > T	p.Ser2309 =	1	
				c.9285C > T	p.Asp3095 =	1	
				c.9396A > G	p.Lys3132 =	1	
				c.10121C > T	p.Thr3374Ile	1	
				c.4584A > G	p.Glu1518 =	1	
				c.8009C > T	p.Ser2670Leu	1	**class 4**

### Cosegregation Analysis

Segregation of 13 VUS was assessed in 11 families (three families carried two VUS, while one VUS was detected in two unrelated kindreds). All the families were of Italian ancestry. Details of the variants, including current classification, *in silico* predictions and cosegregation analysis results are reported in **Table 3**. For 7 variants, cosegregation with the disease in the family was excluded; accordingly, cosegregation ratios in these families ranged from 0.0036 to 0.145.

**Table 3:** Description of families and variants.

Pedigree	Prior probability of pathogenic variant %		Gene	Variant	Class						Predicted effect on splicing	ExAC Allele frequency	Cosegregation				Notes
	BOADICEA	BRCAPRO			LOVD	ENIGMA	ARUP	ClinVar	UMD Share	A-GVGD (GV-GD score)			Y or N	FLB	CSLR	meioses	
281-O-15	BRCA1: 67.4 BRCA2: 3.9	BRCA1: 84.37 BRCA2: 5.63	**BRCA1**	**c.2522G > A p.Arg841Gln**	NC	NR	NP	2	2	C0 (111.49-0.00)	none	0.00004121	N	0.014	0.145	0	*in-trans* with c.5152+2T > G
50-O-14	BRCA1: 0.4 BRCA2: 4	BRCA1: 0.1 BRCA2: 1.3	**BRCA1**	**c.4223A > G p.Gln1408Arg**	NP	NR	NP	3	NP	C0 (29.27-42.81)	none	NP	Y	1.86	1.63	1	
191-O-15	BRCA1: 11.1 BRCA2: 68.1	BRCA1: 3.79 BRCA2: 10.16	**BRCA1**	**c.4895T > G p.Val1632Gly**	NP	NR	NP	NP	3	C0 (262.18-0.00)	none	NP	N	0.0036	0.0099	0	
281-O-15	BRCA1: 67.4 BRCA2: 3.9	BRCA1: 84.37 BRCA2: 5.63	**BRCA1**	**c.5152+2T > G**	NP	NR	NP	NP	NP	NA	donor site broken	NP	Y	1.98	1.86	1	*in-trans* with c.2522G > A
357-O-17	BRCA1: 1.4 BRCA2: 4.1	BRCA1: 0.5 BRCA2: 2.34	**BRCA1**	**c.5509T > C p.Trp1837Arg**	NC	NR	NP	4	5	C65 (0.00-101.29)	none	NP	Y	1.86	1.62	1	
146-O-15	BRCA1: 38.8 BRCA2:1.2	BRCA1: 36.4 BRCA2: 2.8	**BRCA2**	**c.476T > C p.Val159Ala**	NP	NR	NP	3	NP	C0 (170.06-0.00)	none	NP	N	0.0048	0.0284	-1	*in-cis* with c.6290C > T
282-O-17	BRCA1: 1.4 BRCA2: 7.9	BRCA1: 0.31 BRCA2: 0.61	**BRCA2**	**c.1247T > G p.Ile416Ser**	NP	NR	NP	1	3	C0 (353.86-0.00)	none	NP	Y	1.75	1.36	1	
191-O-15	BRCA1: 11.1 BRCA2: 68.1	BRCA1: 3.79 BRCA2: 10.16	**BRCA2**	**c.5386G > T p.Asp1796Tyr**	NP	NR	NP	3	3	C0 (353.86-0.00)	none	NP	N	0.0078	0.0167	0	
368-O-17	BRCA1: 0.3 BRCA2: 2.8	BRCA1: 0.3 BRCA2: 1.36	**BRCA2**	**c.5635G > A p.Glu1879Lys**	NP	NR	NP	2	3	C0 (98.18-0.00)	none	0.0002157	N	0.0321	NA	NA	
275-O-14	BRCA1: 17.4 BRCA2: 2.0	BRCA1: 4.7 BRCA2: 0.8	**BRCA2**	**c.6290C > T p.Thr2097Met**	NC	NR	NP	2	3	C0 (244.82-0.00)	none	0.0001168	Y	1.96	1.94	0	
146-O-15	BRCA1: 38.8 BRCA2: 1.2	BRCA1: 36.4 BRCA2: 2.8											N	0.0048	0.0284	-1	*in-cis* with c.476T > C
18-B-16	BRCA1: 0.8 BRCA2: 6.0	BRCA1: 0.1 BRCA2: 1.2	**BRCA2**	**c.7534C > T p.Leu2512Phe**	NC	NR	NP	3	3	C0 (116.58-8.11)	none	0.00004136	N	0.2	0.77	0	
418-O-17	BRCA1: 0.6 BRCA2: 4.5	BRCA1: 0.99 BRCA2: 0.95	**BRCA2**	**c.7975A > G p.Arg2659Gly**	NP	NR	NP	3	3	C65 (0.00-125.13)	donor site broken	NP	Y	2.63	2.5	2	LOH in OC
115-O-13	BRCA1: 1.7 BRCA2: 0.9	BRCA1: 4.4 BRCA2: 9.0	**BRCA2**	**c.8386C > T p.Pro2796Ser**	NP	NR	NP	2	NP	C0 (145.74-0.00)	none	0.00001647	N	0.11	0.1	0	

*NC, not classified; NP, not present; NR, not reviewed; NA, not applicable; Y, Yes; N, No; FLB, full-likelihood method for bayes factor; CSLR, co-segregation likelihood ratio; LOH, loss of heterozygosity; OC, ovarian cancer*.

### Families Description

**FIGURE 1:**
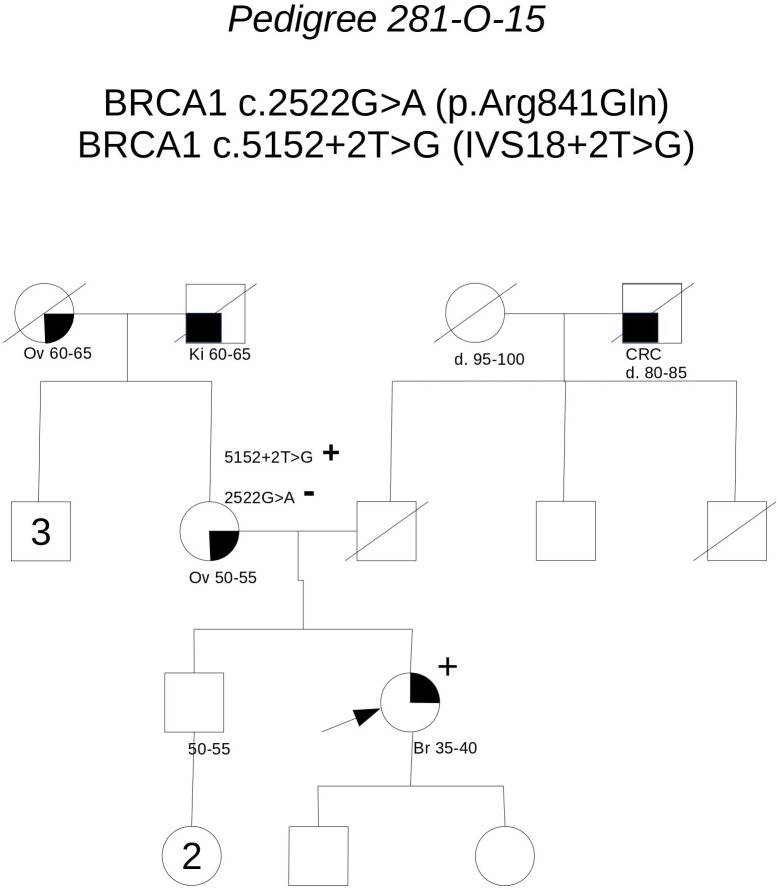
Pedigree of Family 281-O-15 (Br: Breast Cancer. Ov: Ovarian Cancer. Ki: Kidney Cancer. CRC: Colorectal Cancer).

**Pedigree 281-O-15** (**Figure 1**) The proband, a woman aged 50 at the time of counseling, had developed triple-negative breast cancer under the age of 40. Her mother had had surgery for high-grade ovarian cancer in her 50 s and the maternal grandmother was reported with possible ovarian cancer, as well. No significant history of cancer was reported in the father’s side of the family. Genetic testing performed in another center had detected two **BRCA1** variants in the proband: **c.2522G>A** and **c.5152+2T>G**. When she came to our center for a second opinion, we proposed to check the presence of the two variants in the mother: she was found to carry the c.5152+2T>G, not the c.2522G>A variant. This finding, besides supporting the hypothesis that the c.5152+2T>G variant, predicted to affect splicing, may be associated with cancer risk, excludes a role for c.2522G>A in cancer clustering in this family. Moreover, if c.5152+2T>G will be definitely classified as pathogenic in the future, the co-occurrence *in-trans* with c.2522G>A in our patient will provide evidence for conclusively classifying the latter (now class 2) as neutral. Its neutrality is supported by results of functional studies, as no splicing alterations was detected through the minigene assay (Anczuków et al., 2008), and no difference was found, in comparison to wild-type BRCA1, on cisplatin response in a resazurin cell viability assay (Bouwman et al., 2013).

**FIGURE 2:**
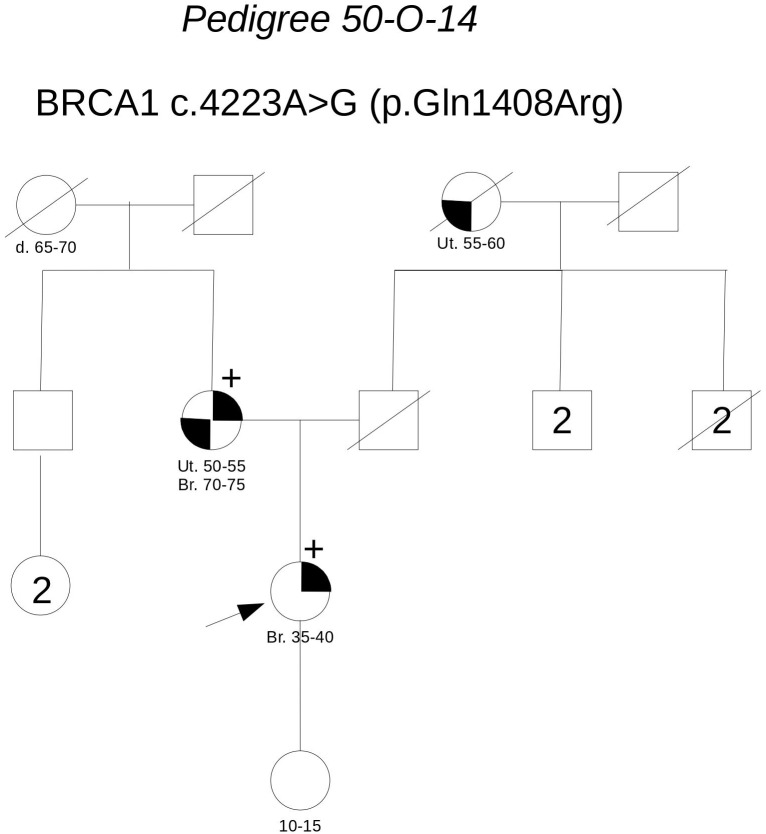
Pedigree of Family 50-O-14 (Br: Breast Cancer. Ut: Uterine Cancer).

**Pedigree 50-O-14** (**Figure 2**) The proband developed hormone-responsive breast cancer under the age of 40 and experienced multisite relapse few years later; her mother, who had undergone hysteroannessiectomy for unspecified reasons, had developed post-menopausal hormone-responsive breast cancer. The **BRCA1** variant **c.4223A>G** was detected in the proband and then confirmed in her mother. This finding failed to provide any significant information on the clinical significance of the variant; however, the low prior probability of BRCA1 pathogenic variants and *in silico* predictions do not support its pathogenicity (**Table 3**). No functional data were available for this variant.

**FIGURE 3:**
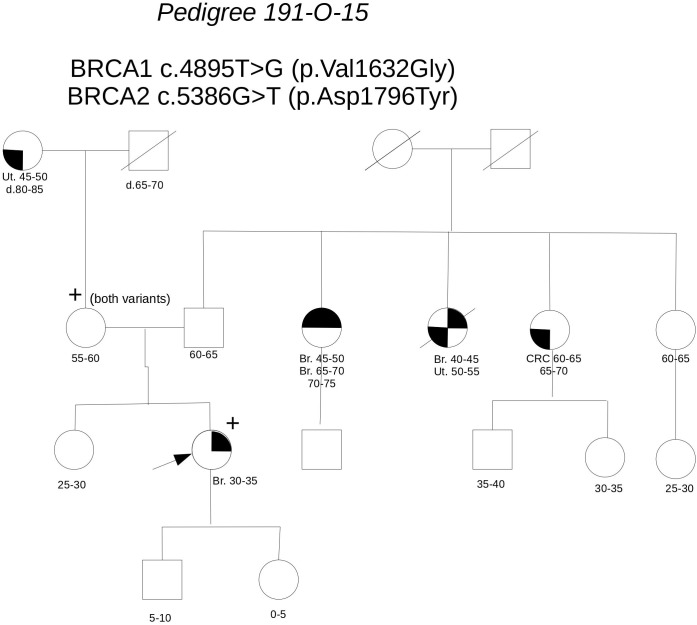
Pedigree of Family 191-O-15 (Br: Breast Cancer. Ut: Uterine Cancer CRC: Colorectal Cancer).

**Pedigree 191-O-15** (**Figure 3**) The proband developed hormone-responsive breast cancer under age 35. Two paternal aunts had breast cancer diagnosed in their 40 s, with one developing contralateral breast cancer over 20 years later. Genetic testing performed in another center in the proband had detected the **BRCA1** variant **c.4895T>G** and the **BRCA2** variant **c.5386G>T**, both reported as class 3 in databases. When she came to our clinic with her mother for a second opinion, we proposed to check the parental origin of the variants by testing the mother, having clinical and family history negative for breast and ovarian cancer. The mother was found to carry both the variants, thus excluding a role for them in breast cancer clustering in the paternal side of the family; together with *in silico* predictions, cosegregation analysis supports the neutrality of both the variants.

**FIGURE 4:**
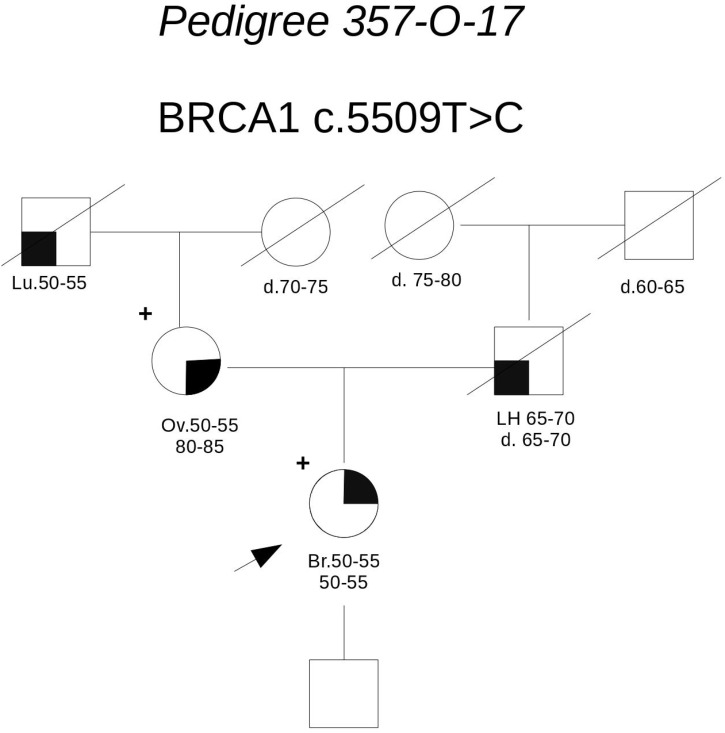
Pedigree of Family 357-O-17 (Br: Breast Cancer. Ov: Ovarian Cancer. Lu: Lung Cancer. LH: Hodgkin Lymphoma).

**Pedigree 357-O-17** (**Figure 4**) The proband had hormone-responsive breast cancer between 50 and 55 years of age. Her mother had developed ovarian cancer at the same age. BRCA testing in the proband led to the detection of the **BRCA1** variant **c.5509T>C**. This variant was subsequently tested in the mother, who was found to carry it, as well. This variant is currently reported as class 5 in the UMD database (class 4 in ClinVar) and its pathogenicity is supported by A-GVGD (C65). Indeed, functional studies have shown this variant to be associated with a severe folding defect, demonstrated through both a protease-based and a peptide-binding assay (Williams et al., 2003, 2004). Therefore, it is confirmed to be pathogenic and to explain the aggregation of breast and ovarian cancer in the family.

**FIGURE 5:**
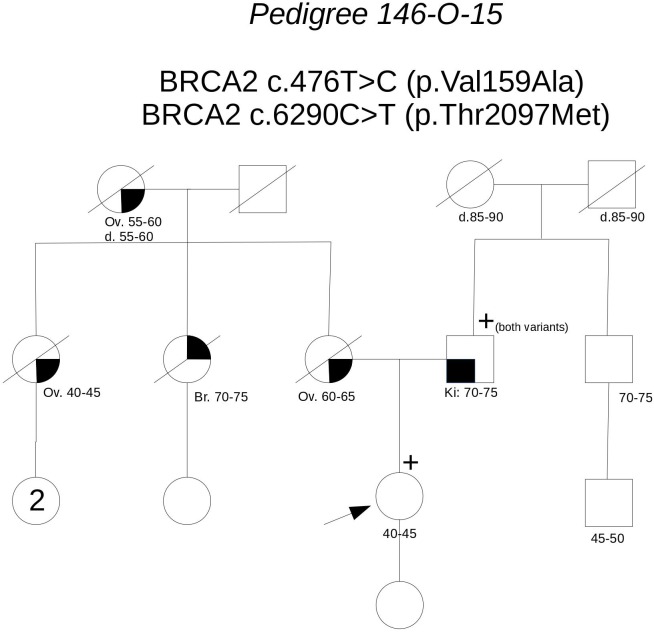
Pedigree of Family 146-O-15 (Br: Breast Cancer. Ov: Ovarian Cancer. Ki: Kidney Cancer).

**Pedigree 146-O-15** (**Figure 5**) The proband is an asymptomatic woman who requested an assessment of her breast and ovarian cancer risk due to a strong history of both malignancies in the maternal side of the family. Indeed, the mother, a maternal aunt and the maternal grandmother had died for ovarian cancer diagnosed between 44 and 65 years of age, the other maternal aunt had a triple-negative breast cancer in her 70 s. Based on her high prior probability of BRCA pathogenic variants (36.4–38.8% for BRCA1; 1.2–2.8% for BRCA2), she was eligible for genetic testing even though she was asymptomatic. Genetic analysis revealed the presence of two **BRCA2** variants: **c.476T>C** and **c.6290C>T**. We proposed to test the father, who was found to carry both the variants. This allowed us to define that the variants were *in-cis* on the allele inherited from the father, thus excluding a role for them in the cancer aggregation in the mother’s side. Together with *in silico* predictions, cosegregation analysis supports the neutrality of both the variants.

**FIGURE 6:**
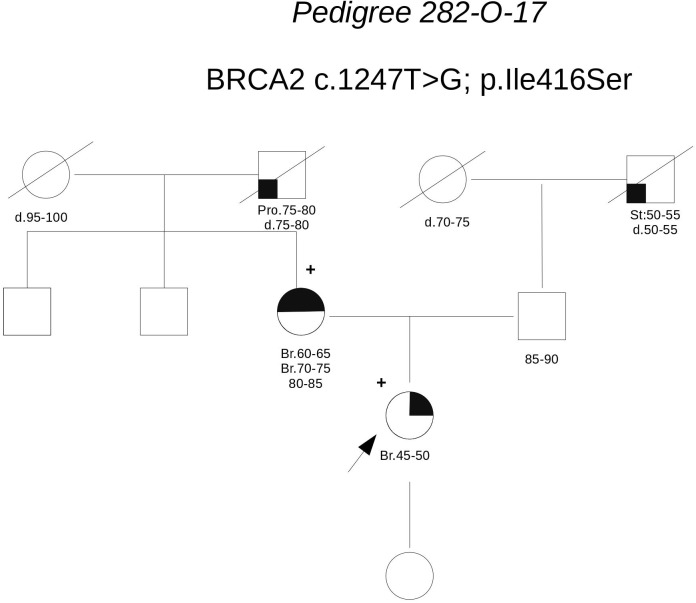
Pedigree of Family 282-O-17 (Br: Breast Cancer. Pro: Prostate Cancer. St: Stomach Cancer).

**Pedigree 282-O-17** (**Figure 6**) The proband developed ductal *in situ* breast cancer under the age of 50 and experienced local relapse (ductal infiltrating carcinoma) some years later; her mother developed hormone-responsive cancer of her right breast in the 7th decade of life and contralateral breast cancer 10 years later. The **BRCA2** variant **c.1847T>G** was detected in the proband and then confirmed in her mother. This finding failed to provide any significant information on the clinical significance of the variant. This finding failed to provide any significant information on the clinical significance of the variant; however, the low prior probability of BRCA2 pathogenic variants in the family and *in silico* predictions do not support its pathogenicity (**Table 3**). No functional data were available for this variant.

**FIGURE 7:**
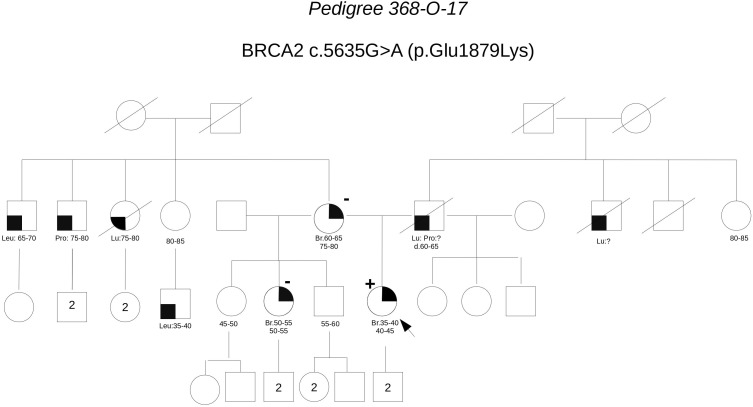
Pedigree of Family 368-O-17 (Br: Breast Cancer. Lu: Lung Cancer. Pro: Prostate Cancer. Leu: Leukemia).

**Pedigree 368-O-17** (**Figure 7**) The proband developed hormone-responsive breast cancer around the age of 40. Her mother had breast cancer in her 60 s. Short after we saw the proband for the first counseling session, a half-sister (same mother), was diagnosed with post-menopausal breast cancer. The proband was found to carry the **BRCA2** variant **c.5635G>A**, which was subsequently excluded in the affected half-sister. One year later, we had the opportunity to analyze also the mother, who tested negative for the variant, thus excluding a role for it in breast cancer clustering in this family. Although no functional data are available for this variant, cosegregation analysis and *in silico* predictions provide data against its pathogenicity.

**FIGURE 8:**
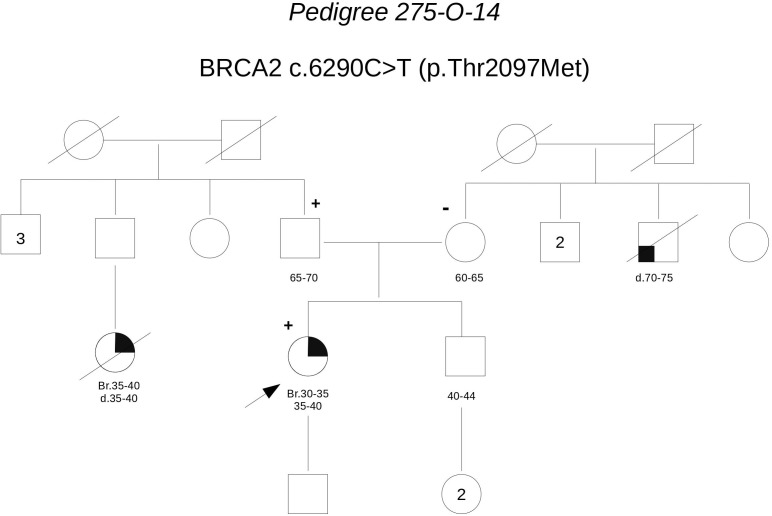
Pedigree of Family 275-O-14 (Br: Breast Cancer).

**Pedigree 275-O-14** (**Figure 8**) The proband developed triple-negative breast cancer in her 30 s. A paternal first-degree cousin was reported to have died for breast cancer diagnosed at a similar age. Genetic testing revealed the **BRCA2** variant **c.6290C>T**. Through testing parents, we could define it had been inherited by the father. However, this finding fails to add relevant evidence on the significance of the variant, which in family 146-O-15 fails to cosegregate with the disease.

**FIGURE 9:**
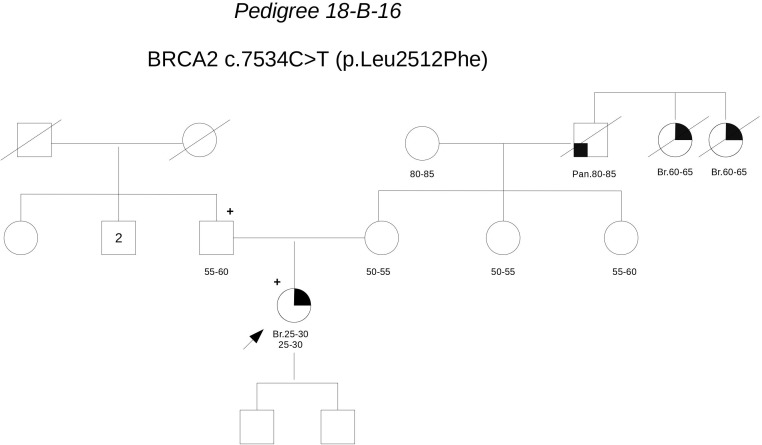
Pedigree of Family 18-B-16 (Br: Breast Cancer. Pan: Pancreatic Cancer).

**Pedigree 18-B-16** (**Figure 9**) The proband developed invasive lobular carcinoma around the age of 30. Her maternal grandfather died for pancreatic cancer and two sisters of him died for post-menopausal breast cancer. Genetic testing detected the **BRCA2** variant **c.7534C>T**. When she came to our clinic with her father for post-test counseling, we proposed to check the parental origin of the variants by testing the father, whose clinical and family histories were negative for breast and ovarian cancer. The father was found to carry the variant, thus excluding a role for them in breast cancer clustering in the maternal side of the family. No functional data are available for this variant, however, cosegregation analysis and *in silico* predictions provide support against its pathogenicity.

**FIGURE 10:**
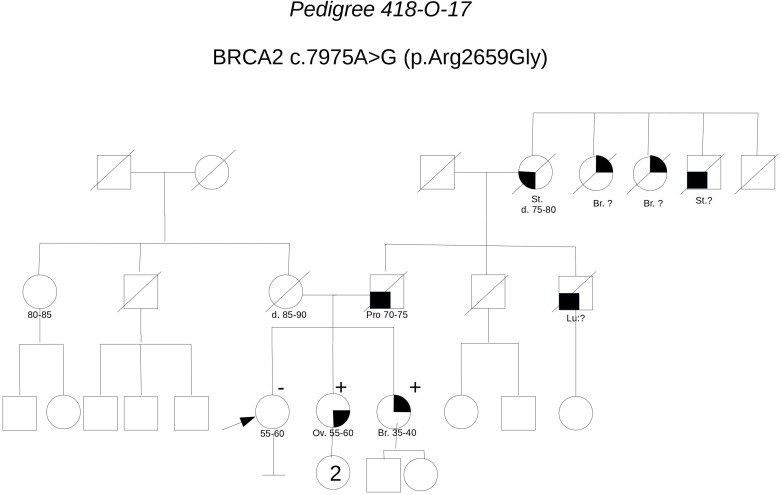
Pedigree of Family 418-O-17 (Br: Breast Cancer. Ov: Ovarian Cancer. Pro: Prostate Cancer. St: Stomach Cancer).

**Pedigree 418-O-17** (**Figure 10**) The proband is an asymptomatic 60-year-old woman who requested an assessment of her breast and ovarian cancer risk due to a history of both malignancies in her sisters. One of the sisters, affected with serous high grade ovarian carcinoma, had BRCA testing performed on cancer tissue in another center, with detection of the **BRCA2** variant **c.7975A>G**. Allelic load in tumor tissue was 80% and the variant was subsequently demonstrated in the germline. *In silico* evaluations supported a pathogenic effect: the amino acid Arg2659, which is substituted by a Glycine residue as a result of the variant, locates in the helical domain just prior to the OB1, with residues in this region fully conserved across all species, including the relatively distant pufferfish, *Tetraodon nigroviridis*. Although c.7975A>G is not described in variant classification databases, other changes of the same amino acid have been classified as definitely pathogenic (class 5). Indeed, the nucleotide c.7975 is located in a consensus splice site and bioinformatic tool predicted splice site alteration. Consistently, both Arg2659Thr and Arg2659Lys have been demonstrated to induce exon 17 skipping in patients’ lymphocytes, (Hofmann et al., 2003; Farrugia et al., 2008). Moreover, allelic load in the tumor suggested Loss-Of-Heterozygosity, thus reinforcing the suspicion. Then, we proposed that the other affected sister, diagnosed with breast cancer in her 40 s, be tested for the variant. The sister was found to carry the variant as well. We then discussing with the proband about the added value of checking whether she carried the variant or not, and she consented to be tested. She tested negative for the variant, which provides additional support to the hypothesis of pathogenicity; as shown in **Table 3**, cosegregation ratio in this family was ≥2 (2–2.63) with all the methods adopted, being the highest in the population under study.

**FIGURE 11:**
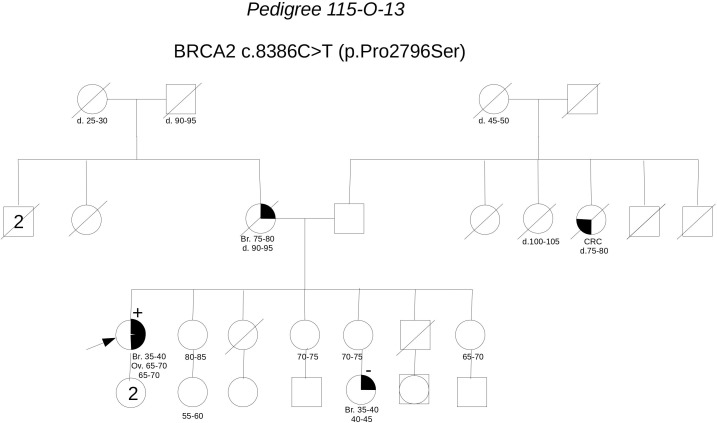
Pedigree of Family 115-O-13 (Br: Breast Cancer. Ov: Ovarian Cancer. CRC: Colorectal Cancer).

**Pedigree 115-O-13** (**Figure 11**) The proband developed breast cancer around the age of 40 and serous ovarian carcinoma 25 years later. Genetic testing revealed the **BRCA2** variant **c.8386C>T**. This variant was subsequently checked in the niece, who had developed breast cancer in her 30 s and was demonstrated not to carry the VUS. This result excluded the variant as a predisposition factor shared by the two women. No functional data are available for this variant, however, cosegregation analysis and *in silico* predictions support its neutrality.

## Discussion

VUSs in BRCA genes are reported in 5–20% of patients undergoing genetic testing (Lindor et al., 2012; Eccles et al., 2015). In line with those findings, we detected class 3 VUS in 6.3% of 1045 breast/ovarian cancer patients analyzed since 2011. Although VUSs are unanimously recognized as seriously challenging risk communication and perception, it is recommended that they are not used for predictive testing in other family members due to their uncertain clinical impact (Plon et al., 2008). Consequently, in most cancer genetics clinics, cosegregation of the variant with cancer in the family is not offered. In addition, quantitative cosegregation analysis performed in a clinical setting is unlikely to provide data significant enough to help classifying a variant, unless it is found in multiple large-size families (Ranola et al., 2018). Accordingly, in our experience, only for one VUS (1673delH in BRCA1), that had been found in 14 families (one very large), cosegregation analysis provided meaningful results to be incorporated in the multifactorial likelihood method, leading to a statistically significant ratio in favor of pathogenicity (Zuntini et al., 2017). All the other VUS were found in 1–5 families each, with pedigree size and structure impairing the significance of a cosegregation analysis. Nevertheless, here we show that cosegregation analysis in selected families may help understand whether that variant may have played a role in cancer clustering in the specific kindred. Indeed, 7 out of 13 variants assessed failed to cosegregate with breast cancer in the family. Although this finding does not allow drawing any definite conclusion on the neutrality of the variant, it may promote a correct perception, by the counselees, about the scarce informativeness of that test result. In fact, many lines of evidence suggest that a VUS result is associated to higher levels of distress, anxiety and risk overestimation, if compared to true uninformative results (Vos et al., 2011, 2012; Culver et al., 2013; Richter et al., 2013). Consistently, bilateral prophylactic mastectomy was performed in 39% of asymptomatic VUS carriers attending the Mayo Clinic; of notice, among the VUSs subsequently reclassified in their experience, 95% were benign (Welsh et al., 2017). Probably, receiving a VUS result has an additive load to risk perception associated to family history: “I and many other women in my family have developed breast cancer AND I carry a BRCA variant: it is definitely genetic.” Excluding that the variant is shared by the other cancer cases in the family is likely to remove a relevant factor of genetic risk overestimation. However, an argument against such a “clinical cosegregation” approach may be that whenever the variant cosegregates with the disease, the false perception that it is causative may be reinforced. Actually, in our sample, among 6 variants cosegregating with the disease, two had additional evidence from literature and *in silico* predictions supporting their pathogenicity. In cases like these, we think that integrating pieces of information regarding the potential pathogenicity of the variant with the specific family situation, where cosegregation further supports its predisposing role, makes the communication process more accurate. To evaluate the actual impact of cosegregation analysis on risk perception, we plan to perform in these patients a qualitative study, using the same methods recently adopted on a different patient sample (Godino et al., 2018).

Finally, it is noteworthy that besides providing information potentially helpful for counseling patients, obtaining cosegregation data and sharing them within the scientific community is crucial to gather significant evidence that may eventually contribute to classify VUSs.

## Ethics Statement

The study protocol conforms to the ethical guidelines of the WMA Declaration of Helsinki and was approved by the Ethical Board of Hospital S.Orsola-Malpighi, Bologna, Italy (Prot. 154/2010/O). All participants gave their informed consent to the analysis and signed the respective form.

## Author Contributions

DT and RZ coordinated the activities, interpreted the results, and draft the manuscript. SF and EB analyzed sequencing data and interpreted the variants. MG and BB managed clinical data, drawn pedigrees, and calculated probabilities of pathogenic mutations. DT, SM, and LG counseled patients, collected informed consents and samples and clinically managed the results. FB performed BRCA genetic testing. All the authors contributed to the manuscript, read and approved the final version of the paper.

## Conflict of Interest Statement

The authors declare that the research was conducted in the absence of any commercial or financial relationships that could be construed as a potential conflict of interest.

## Footnotes

1 http://www.umd.be/

2 http://brcaexchange.org/

3 http://arup.utah.edu/database/BRCA/

4 https://www.ncbi.nlm.nih.gov/clinvar/

5 http://priors.hci.utah.edu

6 https://databases.lovd.nl/shared/genes

7 http://www.umd.be/HSF3/index.html

8 http://rulai.cshl.edu/cgi-bin/tools/ESE3/esefinder.cgi

9 http://agvgd.iarc.fr/

10 https://www.nextprot.org/portals/breast-cancer
